# Mediated configural learning in rats

**DOI:** 10.1080/17470218.2016.1188973

**Published:** 2017-08-01

**Authors:** Tzu-Ching E. Lin, Natasha M. Dumigan, Sergio A. Recio, R. C. Honey

**Affiliations:** a School of Psychology, Cardiff University, Cardiff, UK; b Department of Experimental Psychology, University of Granada, Granada, Spain

**Keywords:** Associative learning, configuration

## Abstract

Three experiments investigated mediated configural learning in male rats. In Experiment 1, after exposure to audio-visual compounds AX and BY, rats received trials where X was paired with shock, and Y was not. The idea that conditioning with X enables the evoked configural representation of AX to be linked to shock received support from the facts that while AX provoked more fear than BX, there was no difference between BY and AY. Similarly, Experiment 2 showed that after exposure to AX and BY, separate pairings of X and Y with shock resulted in more fear to AX and BY than AY and BX. In Experiment 3, rats in group consistent received separate exposures to A and X in Context C, and B and Y in D, while those in group inconsistent received A and X (and B and Y) in both C and D. After rats had received shocks in both C and D, rats in group consistent showed more fear to AX and BY than to BX and AY, but this was not the case in group inconsistent. These results indicate that configural representations, formed either by presenting auditory and visual stimuli as parts of a compound or in a shared context, are subject to a process of mediated learning.

Models of associative learning assume that in order for an association to form between two representations, their corresponding stimuli need to be present in close temporal contiguity (e.g., Rescorla & Wagner, 1972). This assumption is violated by instances of *representation*–*mediated learning* where associations are formed between the memories of stimuli that have been associatively evoked rather than directly activated by their corresponding stimuli. For example, rats given trials on which a tone is first paired with the delivery of food pellets and is then paired with illness induced by lithium chloride, show an aversion to food pellets in spite of the fact that food pellets have never been directly paired with illness (e.g., Holland, 1981). Here, the first stage of training is held to result in the tone coming to activate a representation of food, and when the tone is later paired with illness both the directly activated representation of the tone and the associatively evoked representation of food pellets become linked to illness (for a review, see Hall, 1996). This instance of representation-mediated learning has often been cast in elemental terms, in the sense that the representations that are being (either directly or associatively) activated and paired with illness are held to form separate associations with illness. However, there is also some evidence suggesting that representation-mediated learning can involve evoked configural representations.

Iordanova, Good, and Honey (2008) reported a study in which rats first received presentations of four configurations. For example, they might receive sessions in the morning where a tone was presented in a spotted context, and a clicker was presented in a checked context, and afternoon sessions where the tone was presented in the checked context and clicker in the spotted context. After this training, the tone was paired with shock in a third context (an undecorated test chamber) at midday, and the clicker was not. On the next day, the levels of freezing were assessed in both contexts at both times of day. This assessment showed that rats were more fearful in the context + time of day configurations in which the tone had been presented, the spotted context in the morning and the checked context in the afternoon, than in the other configurations. These results implicate configural processes because the tone had been presented in both contexts and at both times of day, and yet fear was more evident in the context + time of day configurations in which the tone had been presented during the first stage of the study. However, the results do not require that mediated configural learning played a role: The tested configurations might have provoked fear to the extent that they activated the memory of the tone at test rather than because of their similarity to the configural representations (i.e., spotted + morning + tone and checked + afternoon + tone) that had become linked to shock during fear conditioning with the tone. Direct evidence that mediated configural learning was the source of the test results came from a further study that attempted to disrupt mediated learning during the conditioning stage. Iordanova, Good, and Honey (2011) demonstrated that the critical difference in fear to the test configurations was abolished if AP5 (a NMDA receptor antagonist) was infused into the hippocampus during fear conditioning, with the same infusion being without effect when administered during the test itself. Such infusions of AP5 block NMDA receptor-dependent synaptic plasticity in the hippocampus. These results, together with those of various control experiments, implicate a process of mediated configural learning in generating the test results (for a review, see Honey, Iordanova, & Good, 2014).

There is some evidence, from purely behavioural experiments, that is consistent with the view that mediated configural learning is not restricted to configurations involving episodic information (i.e., what happened where and when). For example, in conventional studies of sensory preconditioning, rats might first receive exposure to two flavour “cocktails” (AX and BY) prior to conditioning trials in which X is paired with lithium chloride (and Y is not). This procedure not only results in rats becoming more reluctant to consume a solution containing flavour X than Y, but also results in them being more reluctant to consume a solution containing A than B (e.g., Rescorla & Cunningham, 1978). While this observation might simply reflect the operation of an associative chain at test (with A activating a memory of X, and X activating a memory of lithium chloride) other evidence is inconsistent with this analysis. Thus, when rats receive test trials with AX and BX, they are less inclined to consume AX than BX (see Rescorla & Freberg, 1978; Ward-Robinson, Coutureau, Honey, & Killcross, 2005; see also, Ward-Robinson & Hall, 1996). This finding is inconsistent with the analysis based on an associative chain because the presence of X should mean that both compounds, AX and BX, can directly evoke the memory of lithium chloride. Instead, the fact that AX provokes more conditioned responding than BX suggests that conditioning trials with X had resulted in the evoked memory of AX becoming associated with the memory of illness generated by lithium chloride. This analysis, in terms of mediated configural learning, receives additional support from the results of a recent study by Lin, Dumigan, Dwyer, Good, and Honey (2013).

Lin et al. (2013) gave rats exposure to two audio-visual compounds, AX and BY, prior to presentations of X that were paired with shock and Y that were not. After these treatments, rats showed more fear (less activity) to AX than to BX. Like the results reported by Rescorla and Freberg (1978; see also Ward-Robinson et al., 2005) these findings are consistent with the idea that during conditioning with X the configural representation AX was linked to the memory of shock. However, there is an alternative interpretation for these findings that is based on the fact that the AX compound is familiar whereas the BX compound is not. This difference in familiarity might have been sufficient to generate the observed difference in test performance; for example, because a novel compound (BX) might generate a response that is incompatible with the measured conditioned response (see Honey & Good, 2000; Honey, Good, & Manser, 1998; Honey, Watt, & Good, 1998; see also, Honey, Hall, & Bonardi, 1993). However, this possibility was undermined by a secondary observation from the test stage of the experiment report by Lin et al. (2013). During the test, the novel compound AY was, if anything, more likely to provoke fear than the familiar compound BY. This finding suggests that the familiarity of the test compounds was not the critical factor in determining test performance, and the origin of the difference in fear between AX and BX must lie elsewhere. However, the import of the latter observations is rendered moot to the extent that it relies, at least in part, on the absence of a statistical interaction between the effects at test of the presence of A (versus B) and X (versus Y) in the four test compounds (AX, BX, AY, and BY).

The aim of Experiments 1 and 2 was to investigate mediated learning involving the configural representations of simultaneous audio-visual compounds. Experiment 1 replicated the study reported by Lin et al. (2013), where rats received exposure to AX and BY, then X–shock and Y–no shock trials, and finally test trials with AX, BX, AY, and BY. It also included a statistical analysis that allowed the effects of compound familiarity/novelty to be distinguished from those predicted on the basis of a process of mediated learning. The raw results from Experiment 1 are a subset of those reported in a larger scale study that examined the role of the hippocampus in sensory preconditioning effects (Lin, Dumigan, Good, & Honey, 2016). Critically, Experiment 1 involves a secondary analysis that provides critical information about the role of compound novelty. It also provides an appropriate context for Experiment 2, which used an identical procedure except that the conditioning procedure was changed to permit alternative accounts for the results of Experiment 1 to be dissociated. Finally, Experiment 3 extended the results of Experiment 2 by examining whether the (configural) representations that develop as a result of two stimuli (A and X) being separately presented in the same context (C) are subject to mediated learning in the same way as those formed when they are presented as the components of a simultaneous compound (as in Experiment 2).

## Experiment 1

The experimental design employed in Experiment 1 is shown in Table 1. After exposure to two stimulus compounds, AX and BY, rats received presentations of X that were followed by shock and presentations of Y that were not. During the test in Experiment 1, the fear evoked by four compounds (AX, BX, AY, and BY) was assessed. As we have noted, accounts based on mediated configural learning predict that AX should elicit more fear than BX. To the extent that the difference between AX and BX originates from configural mediated learning involving AX then this effect might be less evident when AY and BY are tested (because AY is less similar to AX than is AX itself). However, if the novelty of the test compounds per se was driving the differences between the familiar AX and novel BX, then this difference should be equally apparent when BY is contrasted with AY. According to this analysis, AX and BY should both evoke more *apparent* fear than should BX and AY.

**Table 1.** Design of Experiments 1–3.

Exposure	Conditioning	Test
Experiment 1		
AX	X→40s→shock	AX versus BX
BY	Y→40s→no shock	BY versus AY
Experiment 2		
AX	X→40s→shock	AX versus BX
BY	Y→40s→shock	BY versus AY
Experiment 3		
Group consistent		
C–A C–X C–A C–X	C→shock	E–AX versus E–BX
D–B D–Y D–B D–Y	D→shock	E–BY versus E–AY
Group inconsistent		
C–A C–X C–B C–Y	C→shock	E–AX versus E–BX
D–B D–Y D–A D–X	D→shock	E–BY versus E–AY

Note: A and B were localized visual stimuli (left and right jewel lights), X and Y were auditory stimuli (tone and clicker), and C, D, and E were experimental contexts; 40s indicates the interval between the presentation of X (or Y) and shock.

### Method

#### Subjects

Eight male naive Lister hooded rats (*Rattus norvegicus*; supplied by Harlan Olac Ltd, UK), with a mean ad libitum weight of 443 g (range: 415–492 g), were used. These rats had received sham surgeries as part of a larger scale study (Experiment 1; Lin et al., 2016). The rats were ≈4 months old at the start of the experiment and were housed in pairs in a colony room that was illuminated between the hours of 8 a.m. and 8 p.m., with food and water available ad libitum throughout the experiment. Behavioural training began at ≈10.00 a.m. on each day. The experimental procedures and animal husbandry conformed to the “principles of laboratory animal care” (Guide for the Care and Use of Laboratory Animals, NIH publication No. 85-23, revised 1985) and the UK Animals (Scientific Procedures) Act (1986).

#### Apparatus

Eight operant chambers (Test chamber 80004-D001; Campden Instruments Ltd., Loughborough, UK; 30.5 cm × 26 cm × 20 cm; width × depth × height) arr-anged in a 4 × 2 array were used. Each had two aluminium side walls, a transparent Perspex back wall, and ceiling. The front wall was also Perspex and served as the door to the chamber. The chambers were housed within sound-attenuating shells, and the chambers were lit by a 3-W light bulb, with a white plastic cover, positioned centrally and 13.5 cm above the floor. Two 30-s visual stimuli served as A and B: the illumination of a left and a right covered 3-W jewel light. These lights, each constantly illuminated throughout their 30-s durations, were mounted 13.5 cm above the floor and were positioned 9.2 cm to the left and right of an unused central wall light mounted at the same height above the floor, but immediately above the food well. Two 30-s auditory stimuli served as X and Y: a 2-kHz tone and a 2-Hz clicker. These stimuli were presented at an intensity of ≈75 dB through a speaker located centrally and at 14.5 cm above the floor on the left aluminium wall, and were produced by an internal audio generator. The ambient noise level was 65 dB. A 0.5-s 0.64-mA shock could be delivered through the grid floor: 19 stainless steel bars with a diameter 0.47 cm and spaced 1.07 cm (centre to centre) apart.

The activity levels of the rats in the chambers were measured using an automated ambulatory monitor that consisted of two horizontal strips, one attached to the front wall (i.e., the door) and the other attached to the back wall. These strips were positioned 3.0 cm above the grid floor and contained three infrared light sources and photo-beam detectors that were located 3.0 cm from the left-hand wall, in the centre of the chamber, and 3.0 cm from the right-hand wall. Detection of the presence of the rat in the area covered by a photo beam followed by detection of the absence of the rat in this area, as the rat moved in the chamber, was recorded as a value of 1. The number of times this occurred for each of the three beams provided a single value for the total movement made by the rat in the chamber. It was assumed that lower levels of activity were indicative of greater fear (subject to appropriate controls). A computer (Mark II Control Unit) controlled the apparatus, ran the experimental software (using Behavioural Net Controller Control 1.0), and recorded ambulatory movements (all equipment was supplied by Campden Instruments Ltd., Loughborough, UK).

#### Procedure

Rats received one exposure session per day for six days (Days 1–6). In each session they received two types of 30-s simultaneous compound: AX (e.g., the left light presented with the tone) and BY (e.g., the right light presented with the clicker). The identity of the visual stimulus that served as A or B, and of the auditory stimulus that served as X or Y, was fully counterbalanced: For half of the rats, the left light served as A, and the right light served as B, and for the remainder the reverse was the case; and within these subgroups half of the rats received the tone as X and the clicker as Y, and for the rest this arrangement was reversed. Each trial type was presented 10 times per session in pseudorandom order with the constraint that there were no more than two trials of the same type in each session. The intertrial interval (ITI) was 2.5 min.

In the conditioning stage, rats received one session per day for two days (Days 7–8). In each session there were three presentations of X (e.g., tone) followed by footshock after a 40-s trace interval and three presentations of Y that were not followed by footshock. For half of the rats, the sequence was XYYXYX, and the rest received YXXYXY with an ITI of 8 min. On the following two test days (Days 9 and 10), rats received four types of 30-s simultaneous compounds: AX, BX, AY, and BY. For half of the rats, Test 1 (involving a comparison of AX versus BX) was on Day 9, and Test 2 (involving AY versus BY) was on Day 10, and for the remainder the assignment of stimulus compounds to Test 1 and Test 2 was reversed. For half of the rats on each day, the order of trials was AX, BX, BX, and AX and for the remainder it was BX, AX, AX, and BX. Similarly, for half of the rats on each day, the order of trials was AY, BY, BY, and AY, and for the remainder it was BY, AY, AY, and BY. The ITI was again 8 min.

The conditioning ratio that was used to gauge the change in fear to X and Y during the brief conditioning stage was calculated in the following way: activity during the final trial of conditioning divided by activity during the first and final trial. Using this ratio, scores approaching zero indicate that fear developed over the course of conditioning. Test performance was also assessed by means of a ratio. To contrast the effects based on test stimulus novelty with those expected on the basis of mediated learning, two test ratios were generated: AX test ratios were derived by dividing activity during the familiar AX compound by activity during both AX and the novel compound BX, and BY test ratios were calculated by dividing activity during the familiar compound BY by activity during both BY and the novel AY compound. When this measure is used, test ratios below .50 indicate that rats are showing more fear (or are less active) during the familiar compounds, AX and BY, than during the novel compounds, BX and AY. In contrast, a mediated (configural) learning effect involving AX (or A) would be evident if the AX ratios were below .50, and the BY ratios were above .50.

### Results and discussion

The mean conditioning ratio was markedly below .50 for X (*M* = .25, *SEM* = .04) but not for Y (*M* = .43, *SEM* = .07). While a paired-sample *t* test revealed that the difference between the ratios was not significant, *t*(7) = −2.15, *p* = .06, *d* = 0.76, 95% confidence interval, CI [−0.06, 1.54], one-sample *t* tests confirmed that the conditioning ratios for X were below chance, *t*(7) = −6.09, *p* < .0001, *d* = 2.15, 95% CI [.83, .44], but that the conditioning ratios for Y were not, *t*(7) = −0.93, *p* > .38, *d* = 0.32. The mean levels of activity (in responses per minute, rpm) on the first X trial (29.75 rpm, *SEM* = 2.35) and Y trial (22.25 rpm, *SEM* = 2.26) did not differ significantly, *t*(7) = 1.03, *p* > .34, *d* = 0.36.

**Figure 1.**
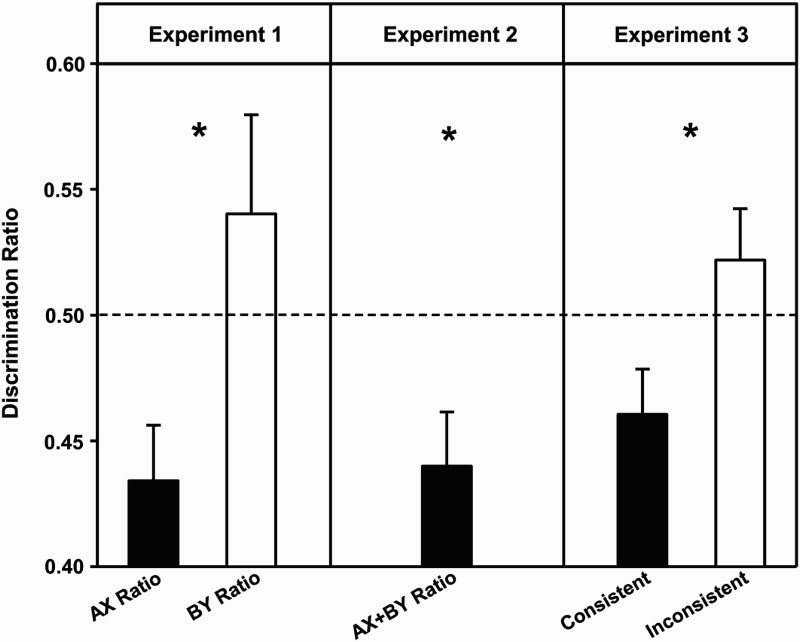
Mean test ratios (+*SEM*) for Experiments 1–3. A score below .50 indicates that the exposed compounds evoked more fear than novel compounds. The asterisks indicate significant differences between the pairs of bars for Experiments 1 and 3, and that the composite bar in Experiment 2 differed from .50. The AX ratios in Experiment 1 and the ratios for group consistent in Experiment 3 also differed significantly from .50.

The critical test results from Experiment 1 are shown in the left panel of Figure 1. The impression gained from inspecting this panel is that while the test ratios for AX were below .5, those for BY were not. A paired-sample *t* test confirmed that the difference between the ratios was significant, *t*(7) = −2.46, *p* < .05, *d* = 0.87, 95% CI [0.02, 1.67]. One-sample *t* tests revealed that while the AX ratios were significantly below .50, *t*(7) = −2.98, *p* < .05, *d* = 1.05, 95% CI [0.15, 1.91], the BY ratios were not significantly above .50, *t*(7) = 1.02, *p* > .34, *d* = 0.36. The mean rates of activity to familiar compounds (AX = 18.71 rpm, *SEM* = 2.79; and BY = 26.00 rpm, *SEM* = 2.51) and the novel compounds (BX = 22.59 rpm, *SEM* = 2.67; and AY = 23.71 rpm, *SEM* = 2.40) did not differ significantly, *t*(7) = −0.41, *p* = .63, *d* = 0.15.

The fact that the exposed compound AX elicited more fear than BX, but the same was not true when comparing the familiar compound BY with AY, indicates that test performance was not determined by the familiarity of the test compounds per se. Instead the finding that AX elicited more fear than BX, but that AY did not elicit more fear than BY, is consistent with the view that mediated configural learning involving AX was the basis of the pattern of test results. However, before accepting this analysis, another related account should be considered. It is also possible that the difference in test performance between AX and BX reflects the fact that X associatively evoked memory of A during conditioning (cf. Holland, 1981; Lin & Honey, 2011). The resulting process of elementary mediated learning could result in greater fear to AX than to BX. According to this account, the finding that the difference in fear between AX and BX was not mirrored in a difference in fear between AY and BY might simply reflect the fact that mediated learning involving A was more evident in the context of a directly conditioned stimulus (X) than during a stimulus that was not paired with shock (Y). Experiment 2 contrasted predictions based on mediated configural and elementary learning.

## Experiment 2

The experimental design used in Experiment 2 is shown in Table 1. The design was identical to that of Experiment 1 with the notable exception that presentations of both X and Y were paired with shock during the conditioning stage. Accounts based on mediated elementary and mediated configural learning make quite different predictions concerning the impact of this simple change. According to the analysis based on elementary mediated conditioning, a representation of A will be evoked on conditioning trials with X, and B will be evoked on conditioning trials with Y. However, the contribution to test performance of mediated conditioning involving A or B should be equally apparent across the four compounds: All contain either A or B. On this basis, the four test compounds (AX, BX, AY, BY) should provoke equivalent fear. However, this prediction ignores the fact that on an AX test trial, X will activate a memory of a stimulus that is physically present (i.e., A), whereas on a BX test trial, X will activate a stimulus (A again) that could provide an additional basis for the BX compound to provoke fear. On this basis, the elementary account predicts that there should be more fear to AY and BX than to AX and BY. In direct contrast, an account based upon mediated configural learning makes the opposite prediction: If conditioning with X and Y allows the evoked representations of AX and BY, respectively, to become linked to the memory of shock, then there should be greater fear (less activity) to both AX and BY than to AY and BX.^1^

### Method

#### Subjects and Apparatus

Sixteen Lister hooded male rats (*Rattus norvegicus*; supplied by Harlan Olac Ltd, UK), with a mean ad libitum weight of 428 g (range: 367–478 g) were used. The rats were ≈4.5 months old at the start of the experiment and were housed in the same manner as rats in Experiment 1, and the apparatus was that used in Experiment 1.

#### Procedure

The rats received an identical procedure to that given to rats in Experiment 1 with the exception that presentations of X and Y were both followed by shock after 40-s trace during the conditioning phase. To be more specific, in the conditioning stage, rats received one session per day for four days (Days 7–10). Rats were given three presentations of X (e.g., the tone) in one session and three presentations of Y (e.g., the clicker) in another session, which were each followed by shock after a 40-s trace interval (i.e., X→shock, Y→shock). For half of the rats, the X→shock trials were on Days 7 and 9, and Y→shock trials were on Days 8 and 10, and, for the remainder, this arrangement was reversed. The ITI was 8 min. Also, during the following two test days (Days 11 and 12), rats received one additional trial with each test compound: AX, BX, AY, and BY. For half of the rats on each day, the order of trials was AX, BX, BX, AX, BX, and AX, and for the remainder it was BX, AX, AX, BX, AX, and BX. Similarly, for half of the rats on each day, the order of trials was AY, BY, BY, AY, BY, and AY, and for the remainder it was BY, AY, AY, BY, AY, and BY. The conditioning ratio took the same form as in Experiment 1, and the test ratio was computed by dividing activity during AX and BY by activity during all four compounds (i.e., AX, BY, AY, and BX). When this measure in used, a score below .50 indicates that rats are showing more fear (less activity) during AX and BY than during BA and AY.

### Results

Over the course of trace conditioning, rats came to show more fear during presentations of X and Y (pooled mean conditioning ratio = .40; *SEM* = .03). A one-sample *t* test confirmed that the scores were significantly below .50, *t*(15) = −2.87, *p* < .05, *d* = 0.71, 95% CI [0.15, 1.25]. The mean level of activity (in responses per minute, rpm) on the first X and Y trial (pooled) was 26.94 (*SEM* = 2.98). The test results from Experiment 2 are shown in the central panel of Figure 1. Inspection of this panel suggests that the test ratios were below .50, which was confirmed by a one-sample *t *test, *t*(15) = −2.79, *p* < .05, *d* = 0.70, 95% CI [0.13, 1.23]. Thus, rats showed more fear to AX and BY than to BX and AY. The mean level of activity (rpm) during AX and BY was 12.35 (*SEM* = 1.60) and during AY and BX was 14.25 (*SEM* = 1.42).

### Discussion

After exposure to AX and BY, conditioning with X and Y resulted in more fear during AX and BY than during BX and AY. This pattern of results is consistent with the results of Experiment 1 and inconsistent with the possibility that mediated elemental learning contributed to differences in test performance. As shown in the introduction to Experiment 2, a simple analysis based on elementary mediated learning predicts that the four test compounds should elicit the same level of fear: Each compound contained an element that was directly paired with shock (i.e., X or Y) and one that could have been associatively activated when shock was delivered (i.e., A or B). However, once it is recognized that AY and BX should also activate the memories of B and A, respectively, then these compounds might be expected to provoke more fear than AX and BY (for which any evoked memories will match those contained in the compound). Clearly, this was not the case. Instead, the results of Experiment 2 provide support for the suggestions that exposure to AX and BY results in the formation of configural representations of these compounds, and that these representations can be activated by X and Y during conditioning and enter into an excitatory association with shock.

## Experiment 3

Taken together, the results of Experiments 1 and 2 provide support for the view that following exposure to a simultaneous compound, AX, pairing X with shock results in the evoked configural memory of AX becoming linked to shock. These results suggest that sensory preconditioning effects involving audio-visual compounds can reflect a process of mediated configural learning (cf. Iordanova et al., 2008, 2011). The issue addressed in Experiment 3 is whether this process plays a role in other phenomena, and, in particular, in demonstrations of acquired equivalence. Honey and Hall (1991) showed that after rats had received presentations of two flavour “cocktails”, AC and XC, pairing X with lithium chloride resulted in rats rejecting A. Like simple instances of sensory preconditioning, this case of acquired equivalence could reflect the operation of a process of mediated elemental learning in which the evoked memory of C becomes linked to the memory of lithium chloride during conditioning with X, with A being rejected at test because it to evokes a representation of C. However, like sensory preconditioning, acquired equivalence might also reflect mediated configural learning. In this case, the presentations of AC and XC might result in the formation of a configural representation (ACX) that is subject to a process of mediated learning during conditioning with X and that mediates generalization to A (see also, Honey & Watt, 1998, 1999). Experiment 3 assessed the merit of this analysis using a version of the experimental design employed in Experiment 2. This experimental design is shown in Table 1.

Rats in group consistent received separate presentations of A and X in Context C and presentations of B and Y in Context D. Thus, A and X were equivalently treated in the sense that they were both paired with C, and B and Y were equivalently treated in the sense that they were both paired with D. The rats were then placed in Contexts C and D where they were given presentations of shock. Finally, the rats received test presentations of AX, BX, AY, and BY in a third context (E). The way in which mediated elemental learning and configural learning might operate in this procedure are described in turn.

According to an elemental analysis, the first stage of training will allow Context C to evoke the memories of A and X and Context D to evoke memories of B and Y. This state of affairs will allow the formation of various elementary associations, most notably between A and X and between B and Y, but also between these elements and C and D, respectively. During conditioning, C and D will become linked to shock, as will the evoked memories of A, X, B, and Y. On an AX test trial, fear will be based upon mediated A–shock and X–shock links and the capacity of A and X to generate a memory of C, whereas on a BX trial fear will be based on mediated B–shock and X–shock links, the capacity of X to evoke A and of B to evoke Y (cf. Experiment 2), and the capacities of X to evoke Context C and of B to evoke Context D. On the basis of the foregoing analysis, and as in Experiment 2, there are grounds to predict that BX will provoke more fear than AX.

Alternatively, presenting two stimuli (e.g., A and X) in the same context (C) might enable the formation of a configural representation (AX or ACX). For example, elemental associations between Context C and A and between C and X will mean that A and X (and C) are activated at the same time, and this might be sufficient to generate a configural representation of AX or ACX. When context C is paired with shock, this will mean that the configural representation activated by C (AX or ACX) will become linked to shock (for a more detailed analysis, see General Discussion). In an analogous way to Experiment 2, this will mean that AX (and BY) will be more likely to elicit fear than BX (and AY).

To rule out the possibility that any difference in fear between the test compounds (e.g., AX and BX) was based upon A and X being presented relatively close together in time (in Context C sessions) and B and Y being presented close together in time (in Context D sessions) a second group of rats was included. Rats in group inconsistent received identical training, with the exception that presentations of A and X (and of B and Y) were presented in both Context C and Context D. For these rats, like those in group consistent, A and X were presented in half of the sessions while B and Y were presented in the remainder. If separate presentations of A and X within one session and B and Y in another were sufficient to allow A and X (and B and Y) to be linked (in some way) then the behaviour of groups consistent and inconsistent should be identical. However, if the consistency of the relationships between the contexts (C and D) and the stimuli (A/X and B/Y) is important, then any differences between the pairs of test compounds (AX/BY and BX/AY) should be evident in group consistent but not in group inconsistent.

### Method

#### Subjects and Apparatus

Thirty-two Lister hooded male rats (*Rattus norvegicus*; supplied by Harlan Olac Ltd, UK), with mean ad libitum weight of 343 g (range = 310–389 g), were used. These rats were approximately 3.5 months old at the beginning of experiment and were housed in the same manner as rats in Experiments 1 and 2. The apparatus was identical to that used in Experiments 1 and 2 with the exception that the upper and lower rows of four boxes were decorated to create two contexts, C and D. Boxes in the upper row were each decorated with laminated spotted wallpaper (diameter: 15 mm; centre to centre distance: 25 mm), and boxes in the bottom row are were decorated with laminated checked wallpaper (30 × 30-mm squares; see Honey & Watt, 1999). This wallpaper was fixed to the aluminium side walls on the inside of the box and was also mounted behind the back transparent Perspex wall on the outside of the box. In this experiment, it was noted that there was very little activity recorded in the zone covered by the left beam and the analysis focused on the centre and right beams.

#### Procedure

All rats received one 30-min exposure session per day for 12 days (Days 1–12). In each of these sessions they received 10 presentations of two 30-s stimuli that were delivered in a pseudo-random sequence in which no more than two trials of the same type could occur in succession. The ITI was 2.5 min. Half of the rats in both groups were placed in Context C on Days 1, 4, 5, 8, 9, and 12, and in Context D on Days 2, 3, 6, 7, 10, and 11, and for the remainder this arrangement was reversed. In group consistent (*N* = 16), when rats were placed in Context C they received presentations of A (e.g., the left light) and X (e.g., the tone), and when they were placed in Context D they received presentations of B (e.g., the right light) and Y (e.g., the clicker). The rats in group inconsistent (*N* = 16) also received presentations of A and X in half of the exposure sessions and B and Y in the remainder, but the two pairs of stimuli were equally likely to be presented in Contexts C and D. Half of the rats in group inconsistent received presentations of A and X in Context C on Days 1, 5, and 9 and in Context D on Days 2, 6, and 10, and presentations of B and Y in Context C on Days 3, 7, and 11 and Context D on Days 4, 8, and 12. For the remaining rats, the days on which they received A and X (and B and Y) in Context C (and D) were reversed. The identities of the context (spotted or checked) that served as C or D, of the visual stimulus (left or right light) that served as A or B, and of the auditory stimulus (tone or clicker) that served as X or Y were fully counterbalanced within each group.

On two of the four conditioning sessions (conducted on Days 13–16) all rats were placed in Context C where they received three shocks, and on the remaining two sessions they were placed in Context D where they also received three shocks. Half of the rats in each group received shock in Context C (e.g., spotted context) on Days 13 and 15 and in Context D (e.g., checked context) on Days 14 and 16, and for the remainder this arrangement was reversed. The inter-shock interval was 8 min.

On the following two test days, rats in both groups received presentations of AX, BX, AY, and BY in Context E (an undecorated test chamber). The compounds were presented in the same way as in Experiment 1. The conditioning ratios used to provide an assessment of the change in activity in Contexts C and D during the conditioning stage were calculated in the following way: activity during the 30-s periods prior to the delivery of the shock on Days 15 (e.g., in C) and 16 (e.g., in D) divided by the sum of activity during the corresponding periods on Days 13, 14, 15, and 16. Using this ratio, scores approaching zero indicate that fear developed over the course of conditioning. The ratios for the critical test took the same form as in Experiment 2 (i.e., activity during AX and BY divided by activity during all four compounds).

### Results and discussion

Over the course of conditioning there was an increase in fear in Contexts C and D in both group consistent (*M* = .28) and inconsistent (*M* = .27). An independent-samples *t* test confirmed that the groups did not differ significantly, *t*(30) = 0.17, *p* > .86, *d* = 0.06, 95% CI [0.03, 0.09]. Two one-sample *t* tests revealed that the ratios in both groups were below .50, smallest, *t*(15) = −4.91, *p* < .0001, *d* = 1.19, 95% CI [0.55, 1.81]. On Days 13 and 14, the mean levels of activity during the first 30-s in Contexts C and D (group consistent: 24.32 rpm; group inconsistent: 22.24 rpm) did not differ significantly, *t*(30) = 0.58, *p* > .57, *d* = 0.21, 95% CI [0.10, 0.31]. Inspection of the right panel of Figure 1 suggests that there was more fear during compounds AX and BY than during BX and AY in group consistent (the test ratios were below .50) and that this was not the case in group inconsistent. An independent-samples *t* test revealed that the test ratios were lower in group consistent than in group inconsistent, *t*(30) = −2.26, *p* < .05, *d* = 0.80, 95% CI [0.40, 1.20],^2^ and one-sample *t* tests confirmed that the test ratios were significantly below .50 in group consistent, *t*(15) = −2.20, *p* < .05, *d* = 0.55, 95% CI [0.01, 1.07], but were not significantly different from .50 in group inconsistent, *t*(15) = 1.07, *p* > .30, *d* = 0.27, 95% CI [−0.23, 0.76]. The overall levels of activity to the two types of test compound (AX and BY versus AY and BX) did not differ between the groups: group consistent (AX and BY = 16.36 rpm, *SEM* = 1.56; and AY and BX = 18.5 rpm, *SEM* = 1.19) and group inconsistent (AX and BY = 16.5 rpm, *SEM* = 1.79; and AY and BX = 15.56 rpm, *SEM* = 1.90), *t*(30) = 0.65, *p* > .52, *d* = 0.22, 95% CI [0.11, 0.35].

The results of Experiment 3 indicate that separate exposure to two stimuli (e.g., A and X) in the same context (e.g., C) produces behavioural effects that match those observed when A and X are presented as the components of a simultaneous compound in Experiment 2.

## General discussion

Mediated learning extends the range of conditions under which associations can form and is a theoretical challenge to models of associative learning (e.g., Rescorla & Wagner, 1972). There has been interest in mediated learning involving the evoked memories of simple, elementary stimuli (e.g., Hall, 1996; Holland, 1981). The experiments presented here assessed whether mediated learning involving configural representations can be observed in rats (cf. Iordanova et al., 2011). Experiment 1 showed that after exposure to AX and BY, pairing X but not Y with shock resulted in more fear to AX than to BX, but no difference in fear between BY and AY. This pattern of results matches that reported by Lin et al. (2013, Experiment 1a) and is readily interpreted in terms of the operation of a process of mediated learning: where AX (or A) becomes linked to shock during conditioning with X. Experiment 2 used an identical procedure to that in Experiment 1, with the exception that now both X and Y were paired with shock during the conditioning phase. There was more fear to the exposed compounds (AX and BY) than to novel compounds that were constructed from familiar components (AY and BX). These findings provide support for the idea that configural representations (of AX and BY) became linked to shock during conditioning with one of their components (X and Y, respectively). In Experiment 3, rats in group consistent received separate presentations of A and X in Context C and of B and Y in Context D, prior to conditioning trials with C and D, and test trials in Context E with AX, BY, AY, and BX. As in Experiment 2, rats showed more fear during AX and BY than during AY and BX. These results suggest that the presentation of two stimuli (e.g., A and X) in a shared context (e.g., C) generates a configural representation that functions in the same way as a configural representation formed as the result of two stimuli being presented as the components of a simultaneous compound (AX). There are two related accounts for how the configural representations were first generated and then entered into association with the memory of shock in Experiment 3 that deserve more detailed consideration. The remainder of the discussion focuses on these accounts.

The first account relies on the assumption that elemental associations form between a given context (e.g., C) and the stimuli presented there (e.g., A and X) during the exposure stage. These associations enable the representations of A and X to be active at the same time, either because both are associatively activated (cf. Dwyer, Mackintosh, & Boakes, 1998) or because one is associatively activated while the other is directly activated (cf. Holland, 1981). In either case, A and X could become linked with the same configural representation (AX) during the exposure stage. According to this account, elemental associations provide the scaffolding upon which the configural representations are constructed. It is then necessary to assume that the resulting configural memory (e.g., AX) can be activated not only by AX during the test, but also by C during conditioning. The general idea that a “simple” association can form between one stimulus (C) and a configuration (AX) is not unprecedented: Theoretical treatments of occasion setting and of discriminative control have relied on a similar idea (e.g., Bonardi, 1989; Bouton, 1990; Holland, 1983). However, this idea leaves the way in which an elemental (C–AX) association activates a specific configuration (AX) somewhat indeterminate.

The second account relies on the idea that exposure to A and X in Context C results in the formation of a configural representation, ACX. This analysis can be aligned to changes in the links between the first two layers of a three-layer network: with input, hidden, and output units corresponding to the elementary stimuli, configural representations, and outcomes, respectively (see Allman & Honey, 2006; Lin & Honey, 2010). Briefly, on first presentation of A in Context C, both would become linked to a configural unit, which would then also become active when X is presented in Context C (by virtue of the presence of C). Under these conditions, each of the elements (A, C, and X) would be capable of activating configural unit ACX. When C is paired with shock, the ACX unit will mediate the association between the input unit X and the outcome (shock), and during the test the presentation of A and X will activate ACX. There are no empirical grounds that allow one to choose between the alternative accounts for how a configural representation involving A and X might have been formed and later activated by C in Experiment 3. However, there is a related body of work, on the acquired equivalence and distinctiveness of cues, that is consistent with an explanation of the results of Experiment 3 in terms of the pattern of links within a three-layer network (Honey & Ward-Robinson, 2002; Honey & Watt, 1998, 1999; Meeter, Shohamy, & Myers, 2009; for a review, see Honey, Close, & Lin, 2010).

The results of Experiments 1–3 provide direct support for the view that patterns of stimulation of relatively simple audio-visual compounds are represented configurally or in the patterns of links within a connectionist network. This is not to say, however, that elementary associations provide no separate contribution to the formation of representations of patterns of stimulation. As already noted, Iordanova et al. (2008) showed that rats form configural memories for patterns of stimulation involving what happened (tone or clicker), where it happened (spotted or checked context), and when it happened (morning or afternoon). However, while mediated learning involving such configural representations was highly sensitive to manipulations that disrupted hippocampal function, effects that only required elemental associations were not disrupted by the same manipulations (e.g., Iordanova et al., 2011; for a review, see Honey et al., 2014). These results provide support for the view that both elemental and configural processes can provide separate contributions to the formation of memories for patterns of stimulation.

In conclusion, the results of Experiments 1–3 indicate that configural representations formed by presenting auditory and visual stimuli either as parts of a compound or in a shared context are subject to mediated learning. The observation that the configural representations, formed in these quite different ways, behave in a functionally equivalent manner raises the question of whether they have a shared origin. Further empirical work will be necessary in order to address this question.

## Disclosure statement

No potential conflict of interest was reported by the authors.

## Notes

1. It is worth noting in passing that should AX and BY evoke more fear than AY and BX during the test in Experiment 2 then this would undermine another potential explanation for the test results of Experiment 1. This alternative is based on the idea that BX might evoke less fear at test than AX because B evokes the memory of a stimulus (Y) that was paired with no shock during conditioning in Experiment 1. This analysis does not apply to Experiment 2 because both X and Y were paired with shock.

2. An independent-samples *t* test revealed that the difference between the test ratios for groups consistent (*M* = .47, *SEM* = .02) and inconsistent (*M* = .52, *SEM* = .02) that was derived from the activity across the three beams approached conventional levels of statistical significance, *t*(30) = −2.05, *p* = .05, *d* = 0.72, 95% CI = [0.36, 1.09].
